# Towards sustainable sanitation management: Establishing the costs and willingness to pay for emptying and transporting sludge in rural districts with high rates of access to latrines

**DOI:** 10.1371/journal.pone.0171735

**Published:** 2017-03-21

**Authors:** Soumya Balasubramanya, Barbara Evans, Richard Hardy, Rizwan Ahmed, Ahasan Habib, N. S. M. Asad, Mominur Rahman, M. Hasan, Digbijoy Dey, Louise Fletcher, Miller Alonso Camargo-Valero, Krishna Chaitanya Rao, Sudarshana Fernando

**Affiliations:** 1 International Water Management Institute, Pelawatte, Sri Lanka; 2 Institute for Public Health and Environmental Engineering, University of Leeds, Leeds, United Kingdom; 3 NGO Forum for Public Health, Dhaka, Bangladesh; 4 Department of Chemical Engineering, Bangladesh University of Engineering and Technology, Dhaka, Bangladesh; 5 BRAC, Dhaka, Bangladesh; 6 Departamento de Ingeniería Química, Universidad Nacional de Colombia, Campus La Nubia, Manizales, Colombia; Boston University School of Public Health, ZAMBIA

## Abstract

**Motivation:**

Proper management of fecal sludge has significant positive health and environmental externalities. Most research on managing onsite sanitation so far either simulates the costs of, or the welfare effects from, managing sludge in situ in pit latrines. Thus, designing management strategies for onsite rural sanitation is challenging, because the actual costs of transporting sludge for treatment, and sources for financing these transport costs, are not well understood.

**Methods:**

In this paper we calculate the actual cost of sludge management from onsite latrines, and identify the contributions that latrine owners are willing to make to finance the costs. A spreadsheet-based model is used to identify a cost-effective transport option, and to calculate the cost per household. Then a double-bound contingent valuation method is used to elicit from pit-latrine owners their willingness-to-pay to have sludge transported away. This methodology is employed for the case of a rural subdistrict in Bangladesh called Bhaluka, a unit of administration at which sludge management services are being piloted by the Government of Bangladesh.

**Results:**

The typical sludge accumulation rate in Bhaluka is calculated at 0.11 liters/person/day and a typical latrine will need to be emptied approximately once every 3 to 4 years. The costs of emptying and transport are high; approximately USD 13 per emptying event (circa 14% of average monthly income); household contributions could cover around 47% of this cost. However, if costs were spread over time, the service would cost USD 4 per year per household, or USD 0.31 per month per household—comparable to current expenditures of rural households on telecommunications.

**Conclusion:**

This is one of few research papers that brings the costs of waste management together with financing of that cost, to provide evidence for an implementable solution. This framework can be used to identify cost effective sludge management options and private contributions towards that cost in other (context-specific) administrative areas where onsite sanitation is widespread.

## Introduction

### Motivation

The benefits of improved sanitation practices to health are well proven [1–3]. There is considerable evidence that the economic benefits of proper sanitation and management of fecal matter are high. In 2012 the World Health Organization (WHO) estimated that a dollar invested in sanitation yields an average benefit of USD5.50 [4]. However, in rural areas of the developing world, improved sanitation has mostly focused on increasing access to (onsite) pit latrines, where the sludge accumulates in a pit in the ground, and stays there. Managing sludge and wastewater from such sanitation structures has received lesser attention.

In recognition of the significant health and environmental risks associated with poor management of fecal sludge and wastewater, target 6.2 of the United Nations’ Sustainable Development Goals calls for universal “access to adequate and equitable sanitation and hygiene for all” where ‘adequate’ ‘[i]mplies a system which hygienically separates excreta from human contact as well as safe reuse/treatment of excreta in situ, or transport to a treatment plant’ [5]. This signals an increased commitment to establishing effective management strategies for treatment of sludge, and not just increasing access to toilets to reduce open defecation. In some locations effective management can be achieved through the use of twin-pit latrines. Each pit is used in turn; once the first pit is full it ‘rests’ until the second pit is nearly full. During this rest period the contents may be partially or completely treated and are then relatively safe to handle. The pit can then be emptied and the contents can then be disposed of or used as a soil conditioner before the first pit is brought back into use. In other cases, pits may be completely abandoned and covered over or used as a planting base for a productive tree, while the super structure is moved. However where space is in short supply or where the cost of twin-pit latrines is viewed to be prohibitive many rural households invest in single pit latrines and these need to be managed in a different way, implying the design of services that collect and transport sludge for treatment.

Designing such a service first requires an estimate of the actual costs of transportation, and the cost per household. One immediate source of monies for financing this cost could be contributions from private households who privately benefit from having sludge removed from their premises (private avoidance benefits) [6]. Households currently make payments to have their pits emptied for continued use, but the sludge is disposed locally [7]. These households might be willing to pay for the costs of collection and transport of fecal sludge for treatment; but this is not well understood. A second source of financing the costs could be through the reuse of fecal sludge for compost or energy. However, recovering this value from sludge in single-pit latrines also hinges on it being collected and transported for processing at a central location, because in-situ recovery from single-pit latrines is challenging.

There is a strong case to be made for understanding the actual costs of transport to a central location for treatment, and the private financial contributions that can be expected from households, or through sale of products produced from sludge. Better management of fecal sludge generates classic externalities—benefits accrue not just to owners of latrines through greater convenience, privacy, improved health and reduced household health expenditure, but to others as well through reduced pathogen load in the environment [8]. The social benefits of managing fecal sludge are higher than the private benefits, implying that left to the market alone, an inadequate level of sanitation services would be provided [9]. From an implementation perspective, identifying the gap between the actual costs and the private contributions is essential before ways to finance this gap (e.g. subsidies or tax concessions to transporting business, charging (or increasing) property taxes, etc.) are considered [10].

Much work has been conducted on the engineering and cost side of waste management at the point of treatment [11–14]. An equally impressive body of work has been conducted on private willingness-to-pay for waste management [15–20]. However, not much work has been done to calculate the actual costs of waste management from onsite latrines in a rural context, or to link the costs of management per household with the households’ willingness to contribute to that cost. This paper uses a methodology that does both, with a view to identifying the gap that needs to be financed. The methodology is employed for calculating costs and the private willingness to contribute to that cost for an administrative area in Bangladesh.

The costs of transporting waste depend on moisture content of the sludge, nature of the terrain, distance to transport, types of equipment used and the method by which waste is transferred from smaller units to larger units within the area over which it is to be transported (see for example [21]). We design and use a spreadsheet-based model to identify a cost-effective option for transporting sludge and use estimates of the capital and operational costs of equipment in common use and distances within the considered administrative area to calculate the per household cost of transporting sludge. We then employ a double-bound contingent valuation method to elicit from households their willingness-to-pay to have sludge transported for treatment, in order to identify the magnitude of private contributions towards the full costs. Contingent valuation is a popular method for eliciting responses on willingness-to-pay for potential goods and services [20, 22–26].

This methodology can be used to identify and operationalize sludge emptying and transport options from onsite latrines in other rural contexts. The spreadsheet model can be used to calculate emptying and transport costs, and the willingness-to-pay methodology can be used to elicit private contribution towards the actual costs in other context-specific administrative areas.

The University of Leeds Faculty of Mathematics and Physical Science, MaPS and Faculty of Engineering joint faculty research committee approved this study. All official and regulatory permissions necessary for conducting research in Bangladesh were coordinated and obtained by NGO Forum for Public Health.

### The context of Bangladesh

In recent years, Bangladesh has made rapid progress in improving sanitation in rural areas and around 40 million new pit latrines have been built. However, this rapid increase in use of household latrines has not been accompanied by a growth in appropriate management of fecal sludge. Currently, when pits fill, they are emptied manually by pit emptiers, who then dispose raw fecal sludge in the vicinity of households [27].

No service (either private, public or ad-hoc) that collects sludge from pits and transports it away for treatment exists for rural Bangladesh. This results in significant increased risk of infection from fecal-oral diseases, including transmission of helminthic infections, to humans; as well as environmental pollution. Though open defecation rates are low at 4% of the population [28] the total economic losses from inadequate sanitation in Bangladesh are estimated to be US$ 4.2 billion each year, equivalent to 6.3% of gross domestic product (GDP) in 2007 [29].

Replacing single pits with toilets that compost in-situ may not be appropriate for Bangladesh, where high moisture content of sludge (> 80%) compromises in-situ composting [27, 30]; where homesteads are often small; and where shallow groundwater is the primary drinking water source [31, 32]. In this context, designing services that manage sludge from pit latrines is important for public health and environmental reasons.

In this paper we estimate the costs of transporting sludge to a central location for treatment, for a subdistrict of rural Bangladesh, the geography at which sludge management is envisaged by the Government of Bangladesh. We also elicit and estimate the willingness of households to pay for collection, and transport away from the home. The costs of operating the treatment process and the revenue from sale of products from treatment (e.g., compost) would be important additional research elements required to design a sustainably financed system but this component is not considered in this paper.

Bhaluka subdistrict in Mymensingh district in central Bangladesh was selected as the site for conducting this research. Bhaluka’s literacy (49.7%), and school attendance rates (47.7%) are slightly below the national average of 51.8% and 52.7% respectively, while urbanization (14.3%) is significantly below the national average of 23.3% [28]. Around 93% of households use a latrine, while 67% use a sanitary latrine (which is most often a pit toilet). The results in this paper are likely to be broadly nationally representative for rural subdistricts of Bangladesh.

## Estimating costs for emptying and transport of fecal sludge

### General approach to cost calculation

We use a spreadsheet based model to estimate the total, per latrine, per emptying event and per household costs of emptying and transporting fecal sludge based on the assumption that the average rate of filling of pits in our earlier study would apply to all pits in the administrative area under consideration [33]. The model takes into account capital costs of equipment, capital maintenance costs, operating costs (including fuel for transport and supplies of safety equipment, cleaning equipment etc.), and labor costs. Several technical options are considered, deploying a range of emptying and transport equipment in various combinations. For both emptying and transport there are potential trade-offs between smaller, manual options and larger, mechanized equipment, which has higher capacity, speed, and range, but also higher capital costs. A range of plausible options was selected based on discussions with operators and a review of equipment available in the market.

The type and quantity of equipment is a function of the amount of fecal sludge which needs to be moved, and the length of time each emptying and transport event takes. The total volume of sludge which needs to be moved to meet 100% of demand for emptying also needs to be estimated. In this paper, the volumes of fecal sludge to be transported in Bhaluka were estimated based on earlier studies reported in [7].

Capital costs, typical capital maintenance replacement periods and estimates of the costs of operational wear and tear were collected from the local market for all the technologies included in the calculation. Unit costs for labor and fuel were also taken from the local market.

Typical performance data for the selected emptying and transport technologies were collected through interviews with operators who have used these technologies in trials locally, and a survey of equipment specifications in the local market. Perspectives on the time needed in each case to empty a typical pit, typical levels of staffing (people per unit) and the time needed to transfer fecal sludge at the point of disposal were also elicited from these operators through interviews and observation. The spreadsheet calculates the total number of emptying events in one year and from this the number of units required to meet 100% of the demand for pit emptying for each combination of equipment modeled.

For ease of comparison the net present value of all costs are calculated assuming an operational period of 25 years. A discount rate applicable to the local context also needs to be selected. In this case, a discount rate of 11% is used; a value of 12% has been recently used in projects in Bangladesh relating to water sector services, which take the social rate of time preference (SRTP) and social opportunity cost (SOC) of capital into account [34].

Total net present value of all costs is then divided by the assumed operational period to give an annualized total cost for each of the technology combinations considered. This can then be divided by the total number of households (to know the ‘per household’ annual cost) or by the number of emptying events for the subdistrict as a whole (to give a cost ‘per emptying’).

### Distance of travel between households and potential treatment sites

Distance of travel has a major influence on total costs. Spatial data on the location of households with latrines, the location of the sites where sludge might be collected and held temporarily (if any) and the location where sludge is finally transported to for treatment need to be collected. Households are usually clustered in villages (or wards) in which case GPS locations of the clusters are assumed to be sufficiently accurate.

In this case, we used spatial data generated from hand-held GPS devices to identify the locations of sampled households in each village in the subdistrict. In each case a central point was then calculated for each village. The location of the treatment facility was assumed to be the subdistrict headquarters. This is normal practice in Bangladesh where municipal solid waste treatment, for example, is concentrated in subdistrict headquarters. The union is potentially a place where sludge could be held temporarily before it is finally transported to the subdistrict headquarters for treatment. The location of each union headquarters and the subdistrict headquarters was obtained from government records. As a general rule we assumed that the routes between villages and union headquarters were non-metaled tracks, whereas the routes between union headquarters and subdistrict headquarters were assumed to be metaled roads. This assumption is rather conservative in terms of calculating transport costs, as many villages are at least partly connected to union headquarters by means of metaled roads. Total travel distances, on both types of road, were then calculated for each village in each union using the GPS coordinates collected earlier. These were then weighted for population, summed, and then divided by the total number of villages to give the average travel distance by track and road between each village and its union headquarters and each union headquarters and the subdistrict headquarters.

### Selection of technologies for emptying and transport

A review of the opinions of local pit emptiers and global sanitation experts was conducted to identify locally feasible technologies for emptying and transporting sludge. Key informant interviews, and online surveys are some of the tools that can be employed to collect the data needed for identifying suitable technologies. These technologies, especially simple pumps, and simple transporting options, could then be tested in the field to gather data especially on labor and time requirements.

In this case, a literature review, a survey of 31 international sanitation experts, interviews with local pit emptiers, and in-field testing of a few pumps and simple transportation options was used to identify that the most suitable improved emptying technology for rural Bangladesh, including for our study site, was the diaphragm pump, a hand held non-powered tool which enables the removal of sludge and wastewater from pits while minimizing human contact [27]. Currently the vast majority of pits are emptied manually without the use of mechanized equipment of any kind (data obtained from the household survey described in the section on willingness to pay below). We therefore selected two options for modeling the costs of emptying; manual emptying and the diaphragm pump. For transport we considered the use of small trucks carrying plastic barrels which is the most common method currently used by pit emptying contractors in rural Bangladesh. These trucks can usually access villages even where metaled roads are not available. Usually pit contents are transported to the place of disposal using these trucks, but we also considered the additional use of tankers with a typical capacity of 15,000 liters to transport fecal sludge between union and subdistrict headquarters. The use of this additional transport option would require transfer of material from trucks to the tankers at the union headquarters, probably at a simple transfer station consisting of ramp and a tank. We did not include the costs of transfer stations, which are considered to be minimal compared to the operating costs of the system as a whole. A summary of the options considered is shown on Table 1.

**Table 1:** Options for modelled costs.

Option	Method of Emptying	Transport	
		Village to Union	Union to Upazila
1	Manual	Trucks	Trucks
2	Manual	Trucks	Tanker
3	Diaphragm Pump	Trucks	Trucks
4	Diaphragm Pump	Trucks	Tanker

### Results

#### Typical travel distances

Average travel distances for each union are shown in Table 2. Overall the average distance between a village and the union headquarters was found to be 3.5 km and the average distance from a union headquarters to the subdistrict headquarters was 8.9 km.

**Table 2:** Travel distances in Bhaluka subdistrict.

Union		Average Travel Distance by Union (km)		
	No. of villages	Village to Subdistrict HQ	Village to Union	Union to Subdistrict HQ
Bhaluka	6	2.7	2.7	0.0
Birunia	12	16.4	7.2	9.2
Dakatia	10	15.5	3.2	12.3
Dhitpur	6	8.4	2.1	6.3
Habirbari	7	10.7	2.4	8.3
Kachina	11	21.2	4.9	16.3
Meduary	11	9.7	3.0	6.7
Mallikbari	11	9.6	3.5	6.2
Rajai	10	11.6	2.5	9.1
Uthura	7	13.9	1.6	12.3
Bharadoba	4	5.8	1.8	4.0
Bhaluka Upazila Average		12.4	3.5	8.9

#### Costs and performance characteristics of emptying and transport equipment

Unit costs of labor and fuel are summarized in Table 3. The cost and performance characteristics of the two emptying options and the two transport options are summarized in Tables 4 and 5 respectively.

**Table 3:** Unit costs and working hours.

Element	Unit	Rate
Wages		
Skilled Labor (Pit Emptying)	BDT/hour	20
Skilled Labor (Transportation)	BDT/hour	20
Unskilled Labor	BDT/hour	15
Working hours		
Average Hours worked per Day	hours	8
Average Days Worked Per Week	days	4
Average Operational Weeks Per Year	weeks	40
Average Operational Hours per Year	hours	1,280
Fuel costs		
Fuel Costs	BDT/litre	68

Source: Pit emptying survey and authors' calculation

**Table 4:** Performance characteristics-emptying options.

Element	Unit	Manual	Diaphragm
Preparation time	Minutes	30	15
Emptying time for full pit	Minutes	150	20
CAPITAL COSTS			
Capital Costs	BDT	1,000	30,000
Capital replacement period	Years	3	5
Capital maintenance cost	% capital costs	100%	100%
Operational wear and tear	% capital costs/year	10%	15%
Annualised capital costs	BDT/year	156	3,909
OPERATIONAL COSTS			
Nr of skilled operators		2	1
Nr unskilled operators		1	
Labour costs	BDT/hour	55	20

Source: Market survey and authors' calculation

**Table 5:** Performance characteristics-transport options.

Element	Unit	Truck	Tanker
Loading/Emptying Time	Minutes	30	60
Travel speed (Tracks)	km/hour	10	
Travel Speed (Roads)	km/hour	30	30
Capacity	litres	120	15000
CAPITAL COSTS			
Capital cost	BDT	624,000	1,560,000
Capital maintenance/ replacement period	Years	7.5	10
Capital maintenance cost	% capital costs	100%	100%
Operational wear and tear	% capital cost/year	10%	15%
Annualised capital costs	BDT/year	46,124	154,003
OPERATIONAL COSTS			
Wages			
Nr of skilled operators		1	1
Nr unskilled operators		1	1
Labour costs	BDT/hour	35	35
Other operational costs			
Fuel consumption	litres/km	0.1	0.2
Other consumables	BDT/km	0.1	2
Operational costs (excluding labour)	BDT/km	6.9	15.6

Source: Market survey and authors' calculation

#### Total costs for emptying and transport of fecal sludge for Bhaluka Subdistrict

There are a total of 77,413 households with single pit latrines in Bhaluka [28]. Although the average household size is 4, the average number of users of each latrine is 5 due to shared use of approximately 20% of the household latrines. The pits have on average 2.4 rings, with a diameter of 0.83m. The typical sludge accumulation rate in Bhaluka is calculated at 0.11 liters/person/day [27]. This means that the typical latrine will need to be emptied approximately once every 3.7 years. For the whole population, that results in the need for 20,760 emptying events each year, and a total of 15,219 m^3^ of sludge to be emptied and transported annually.

The four options for managing emptying and transport of all this fecal sludge are summarized in Table 6. To empty all pits as they fill requires 49 teams of manual emptiers or ten teams of emptiers equipped with a diaphragm pump—i.e., considerably more than are currently operating in the area. Transport would require 228 Trucks, or 169 trucks and 3 large tankers, if transfer to larger tankers were an option. Between 354 and 603 jobs would be created or maintained depending on the technology option selected.

**Table 6:** Summary of emptying and transport solutions.

Option	Emptying		Transport				Total staffing (people)
	Method	Nr of Units			Nr of units		
			Village to Union	Union to upazila	Trucks	Tankers	
1	Manual	49	Trucks	Trucks	228	-	603
2	Manual	49	Trucks	Tanker	169	3	491
3	Diaphragm Pump	10	Trucks	Trucks	228	-	466
4	Diaphragm Pump	10	Trucks	Tanker	169	3	354

Source: Authors' calculation

Table 7 summarizes the total costs of each option. The least expensive option is option 4 (use of diaphragm pumps and a combination of trucks and tankers) with an annual cost of BDT 22.6 million (USD 289,444). The most expensive option is option 1 (manual emptying and trucks only) with an annual cost of BDT 45.8 million (USD 586,845).

**Table 7:** Summary of costs.

Option	Total capital costs	Annual cost ^1^^,^ ^2^						Monthly cost per household with a pit
		Annualised capital costs	Labour	Operating costs	Total	Cost per emptying event	Cost per household with a pit	
BDT								
1	142,321,000	10,523,891	13,595,855	21,654,197	45,773,943	2,205	591	49
2	110,185,000	8,264,596	11,061,555	6,402,234	25,728,385	1,239	332	28
3	142,572,000	10,555,314	10,412,666	21,654,197	42,622,178	2,053	551	46
4	110,436,000	8,296,020	7,878,366	6,402,234	22,576,620	1,088	292	24
USD ^3^								
1	1,824,628	134,922	174,306	277,618	586,845	28	8	0.63
2	1,412,628	105,956	141,815	82,080	329,851	16	4	0.36
3	1,827,846	135,325	133,496	277,618	546,438	26	7	0.59
4	1,415,846	106,359	101,005	82,080	289,444	14	4	0.31

Source: Authors' calculation

^1.^Annualised capital costs includes cost of servicing capital, capital maintenance and operational wear and tear, over the operational period which is 25 years

^2.^ Operating costs includes fuel, and other operational supplies

^3.^ 1USD = BDT78

These total costs are equivalent to a cost of between BDT 1,088 and 2,205 (USD 14 to 28) per emptying event or BDT 24 to 49 (USD 0.31 to 0.63) per household per month if each household in the subdistrict paid a regular tariff for ongoing pit emptying services.

**Fig 1:**
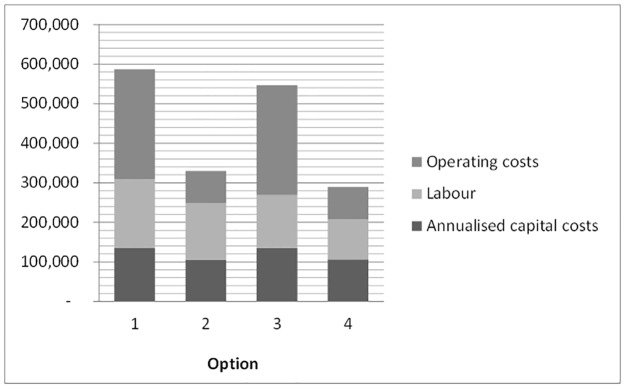
Breakdown of annualized costs for each option (in USD).

The detailed breakdown of annualized total costs for each option is shown in Fig 1. Options 1 and 3, using trucks only, have higher total costs, due primarily to the high operating costs, comprising fuel used in multiple journeys carrying small volumes of sludge. Transferring fecal sludge to larger tankers at the Union headquarters level (Options 2 and 4) significantly reduces the costs of transport. Comparing Options 2 and 4 shows that the use of diaphragm pumps marginally reduces costs, primarily due to time and therefore labor savings. Although the diaphragm pump has higher capital costs compared to manual equipment, the latter must be replaced more often, and more units are required to service the same number of toilets. Diaphragm pumps also offer considerably greater protection to the health of operators and are therefore recommended in preference to manual emptying [27].

## Contributions from the private purse: Pit latrine owners’ willingness-to-pay for a collection-and-transportation service

### A model for estimating willingness to pay using dichotomous choice with follow-up

Contingent valuation can be used to estimate willingness-to-pay (WTP) for a prospective collection and transportation service (simply referred to as ‘service’ from now on). Contingent valuation is a survey based methodology for eliciting values people place on goods, services, or amenities for which direct markets don’t exist, or when revealed preference methods are difficult to apply [35]. This method can be used to value one or more good or service, or an attribute.

In this paper, a dichotomous choice (DC) with a single follow-up question was used. The respondent was read a description of the prospective service, presented with a base bid which was determined by the researcher, and asked if s/he is willing to pay the base bid for the service described (a yes/no response). If the respondent accepts the base bid, s/he was presented with a follow-up bid higher than the base bid, and asked if s/he is willing to pay that bid. If the respondent rejects the base bid, then s/he was presented with a follow-up bid lower than the base bid. Both higher and lower bids were also determined by the researcher. Every respondent was presented with two bids—a base bid, and a follow-up bid that depends on accepting or rejecting the base bid.

Contingent valuation methods can be implemented in alternate ways and the choice is often context-specific. DC methods are considered more compatible for truthful revelation of preferences than open-ended or payment card methods [36]; the follow-up question provides more information for estimating WTP [37]. On the other hand, DC methods are subject to the problem of anchoring [38]; which might be exacerbated when there is a follow-up question [39]. In this case, the researchers had a reasonable prior estimate for the expected mean or median WTP, enabling the construction of bids around these measures of central tendency [40–43].

Let the WTP be modelled as a function, where:

$$WTP_{i}\left(x_{i,},\;e_{i}\right)=\;x_{i}\beta +e_{i}$$ (1)

In Eq (1), *x*_*i*_ is a vector of explanatory variables that include the individual’s characteristics, as well as the two bids presented to it; *β* is a vector of parameters that describe the effect of the individual’s, characteristics and the presented bids on the WTP. *e*_*i*_ is the error term such that *e*_*i*_ ~ *N*(0, *σ*^2^). Let *b*_1_ be the first bid that the respondent is presented with, and *b*_2_ the next bid. Let $y_{i}^{1}$ and $y_{i}^{2}$ be dichotomous variables that denote the responses of the individual *i* to the first and second bid, respectively. Following [44] there are four possible outcomes:

- $y_{i}^{1}$ = 1 *and* $y_{i}^{2}$ = 0; *then*, *b*^1^ ≤ *WTP* < *b*^2^. *Thus*:
  $$\text{Pr}\left(s,n\right)=\Phi \left(x_{i}^{\prime }\frac{\beta }{\sigma }-\;\frac{b^{1}}{\sigma }\right)-\;\Phi \left(x_{i}^{\prime }\frac{\beta }{\sigma }-\;\frac{t^{2}}{\sigma }\right)$$ (2)
- $y_{i}^{1}$ = 1 *and* $y_{i}^{2}$ = 1; *then*, *b*^2^ ≤ *WTP* < ∞. *Thus*:
  $$\text{Pr}\left(s,s\right)=\;\Phi \;\left(x_{i}^{\prime }\frac{\beta }{\sigma }-\;\frac{b^{2}}{\sigma }\right)$$ (3)
- $y_{i}^{1}$ = 0 *and* $y_{i}^{2}$ = 1; *then*, *b*^2^ ≤ *WTP* < *b*^1^. *Thus*:
  $$\text{Pr}\left(s,n\right)=\;\Phi \left(x_{i}^{\text{'}}\frac{\beta }{\sigma }-\;\frac{b^{2}}{\sigma }\right)-\;\Phi \left(x_{i}^{\text{'}}\frac{\beta }{\sigma }-\;\frac{b^{1}}{\sigma }\right)$$ (4)
- $y_{i}^{1}$ = 0 *and* $y_{i}^{2}$ = 0; *then* 0 < *WTP* < *b*^2^. *Thus*:
  $$\text{Pr}\left(n,n\right)=1-\;\Phi \;\left(x_{i}^{\prime }\frac{\beta }{\sigma }-\;\frac{b^{2}}{\sigma }\right)$$ (5)

The parameters *β* and *σ* are estimated by maximizing the following likelihood function (7) for all respondents where:

i ∈(1, N) (6)

$$\sum_{i=1}^{N}d_{i}^{sn}ln\left(\Phi \;\left(x_{i}^{\prime }\frac{\beta }{\sigma }-\;\frac{b^{1}}{\sigma }\right)-\;\Phi \left(x_{i}^{\prime }\frac{\beta }{\sigma }-\;\frac{b^{2}}{\sigma }\right)\right)+d_{i}^{ss}ln\left(\;\Phi \;\left(\;x_{i}^{\prime }\frac{\beta }{\sigma }-\;\frac{b^{2}}{\sigma }\right)\right)+\;d_{i}^{ns}ln\left(\Phi \;\left(x_{i}^{\prime }\frac{\beta }{\sigma }-\;\frac{b^{2}}{\sigma }\right)-\;\Phi \left(x_{i}^{\prime }\frac{\beta }{\sigma }-\;\frac{b^{1}}{\sigma }\right)\right)+d_{i}^{nn}ln\left(\;1-\Phi \;\left(\;x_{i}^{\prime }\frac{\beta }{\sigma }-\;\frac{b^{2}}{\sigma }\right)\right)$$ (7)

where the variables *d*_*i*_^*sn*^, *d*_*i*_^*ss*^, *d*_*i*_^*ns*^ and *d*_*i*_^*nn*^ are indicator variables that take the value 1 or 0; thus an individual contributes to the likelihood function in only one of its four parts [44]. Once $\hat{\beta }$ and $\hat{\sigma }$ are estimated, the WTP is calculated as $\hat{WTP}=\;x^{\prime }\hat{\beta }$.

### Describing the service and constructing bids

A simple description of the service that would transport the sludge away for treatment needs to be presented to the respondent. Then the bids need to be constructed. The bids are typically set keeping in mind the market price of an existing service that might be an imperfect substitute to the hypothetical one being described, or keeping in mind the price of the currently prevailing practice of managing sludge used by households. Surveys of pit latrine emptiers were used to gain an understanding of these charges, in order to construct the bids. The contingent valuation study needs to be conducted on households with pit latrines, in the same administrative area for which the costs of transportation are being estimated.

In this paper, the WTP study was conducted in Bhaluka, to enable comparisons to the costs of the service previously calculated. The current practice of using professional pit emptiers, who empty the pit during nighttime using bare hands and buckets and dump it in the backyard of the homestead in a shallow trough [27], is called ‘empty-and-dump’. It can be regarded as an imperfect substitute to a collection-and transportation service: both methods continue to provide the household with a functional toilet when the pits fill, but the proposed service removes sludge from the premises, providing additional private aesthetic benefits. It can be argued that the private benefits accruing to the household from the service are at least as high, if not higher, than that from the empty-and-dump practice.

Thus, the following service was described to the respondent:

“Please think about the pit latrine that was built for you. When the pit is full, you will probably hire a pit emptier to empty it. You would be able to continue using the latrine, but the waste would be dumped in a shallow trough near your house, or be thrown into a water body if your house is next to one. However, if a service were designed to collect and transport sludge, you would be able to continue using your latrine, and the waste would be taken far away from your house. You may have to spend a bit more money, but the backyard of your house would be clean and not polluted. We are interested in understanding if you value the waste from your pit latrine being taken far away from your house.”

Since empty-and-dump is an imperfect substitute for the service, focus group discussions were held with pit emptiers to understand how charges were levied currently, in order to construct bids for the service. Most pits have the same diameter, thus variations in volume are mostly attributed to the depth. Charges for empty-and-dump were rather flat, and did not vary much with the volume of the pit, largely because the amount of time involved in opening the pit and preparing the shallow trough for dumping is independent of the volume of the pit. Emptiers reported charging ~BDT 400 for empty-and-dump.

With a mechanized collect-and transport service, the total amount of time taken to empty the pit would vary even less with its volume, while delivering additional private benefits. Thus bids for the hypothetical service were constructed using reported charges for empty-and-dump, increasing the charges slightly to capture additional private benefits from the service. Four base bids, set at BDT 400, 600, 700 and 800 were determined. Table 8 displays the base bids, and the follow up bids associated with the base bids. If a households assigned a base bid of BDT 400 rejected it, it was presented with a follow-up bid of BDT 300. In cases where the base bid was set at BDT 800 and the household accepted it, it was presented with a follow-up bid of BDT 1,000.

**Table 8:** Vector of bids and number of households.

	Follow up bid		
Base bid	Base bid response = yes	Base bid response = no	No. hhs
400	600	300	286
600	700	400	280
700	800	600	282
800	1000	700	243

### Sampling strategy

Sample size calculations need to conducted, in a manner relevant to the administrative area in which the study is being designed. This is done to ensure that the sample has sufficient statistical power. A list of households owning pit latrines also needs to be procured to enable the selection of a random sample. If households within the administrative are clustered into villages or wards, then a two-step random sampling process that first selects clusters, and then households in the cluster, is recommended. Sampling weights may have to be constructed to improve the ability of the sample to make predictions about the population; and standard errors may need to be clustered at the cluster level.

In this paper, it was assumed that 50% of the households in Bhaluka had emptied their pits at least once, while the rest were yet to empty. A confidence level of 95%; a design factor of 2 to account for village-level clustering; and a non-response contingency of 5%, was considered. The minimum sample size for this study was calculated as 807 households. To increase the statistical power of the test the sample size was increased to 1,091 households (approximately 1% of rural households in Bhaluka).

While latrine coverage in Bhaluka is impressive census data cannot be used to identify households with pit latrines. Since the purpose of this study was to understand WTP for emptying and transporting sludge from pit latrines, we needed to select a sample where all households own a latrine. BRAC, a major agency responsible for the installation of ~10,000 pit latrines in Bhaluka, maintains a list of households whose pits were installed by the program, along with their addresses. This list was used to select a random sample of households.

A two-step random sampling rule was used. Villages with less than 15 BRAC-financed single pit latrines were excluded. A village was randomly selected (without replacement) and 40% of BRAC households in that village were sampled. This was done till the 1,091 households were selected. Thus, our sample consists of 1,091 households in 44 villages.

In our sample, the probability of a household living in a village with fewer BRAC households being selected into the sample is higher than that for a household living to a village with more BRAC households. Sampling weights are used to address the unequal probabilities of selection, and are calculated as the inverse of the product of two probabilities: the probability of a village being selected, and the probability of a BRAC household within that village being selected. Finally, all results are reported after accounting for clustering at the village level to ensure that the estimates are not mistaken to be more precise than they actually are.

Since BRAC households may be different from non-BRAC households, the asset register, a self-reporting record, maintained by the Bhaluka subdistrict government office was used to procure a list of non-BRAC households with pit latrines in the 44 villages in our sample. Using a similar two-step sampling process, 15 villages from each of the 44 villages were randomly chosen, amounting to 660 households. Analysis was conducted on this sample, and household characteristics, revealed and stated behaviors, as well as willingness-to-pay for the service were found to be similar. We do not include data from non-BRAC households in this paper.

### Data and methods

Each respondent in the sample was randomly assigned to receive a vector of bids to respond to. A questionnaire was designed to elicit responses to the vector of bids, and to elicit other socioeconomics data.

In this case, a questionnaire was implemented to each BRAC household in which pit emptying behaviors and payments for empty-and dump were elicited. For the willingness-to-pay question, within each village, selected households were randomly allocated to receive one of the four vectors of bids in Table 8. Of the 1,091 households, two hundred and eighty six households were randomly assigned to receive BDT 400 as the base bid, 280 were assigned to BDT 600 as the base bid, 282 to BDT 700, and 243 to BDT 800.

We also elicited from the respondents, their perceptions about alternative pit emptying and sludge disposal practices. Respondents were given ten stones and a cup, and were asked to express the degree of their agreement to a statement by putting marbles in the cup [45]. Perceptions related to cleanliness, safety and convenience of empty-and-dump and collect-and-transport service were elicited. Information on household characteristics was also collected. The questionnaires were administered to the male head of the household.

### Results

#### Summary statistics

In our sample, the average household size was 4.56 (Table 9). In 50% of households, the highest level of education was secondary school (Class 6–10), while in 34% of households, primary education was the highest education level. The mean annual income per household was USD 1,246, amounting to approximately USD 2.8/ day in 2005 purchasing power parity. This is just over twice the World-Bank defined international poverty line of USD 1.25/day, in 2005 purchasing power parity. Around 66% of households are involved in non-farm occupations, while a similar percentage report living in houses with brick walls. Metal sheets were the most common roofing material. Comparing these statistics to the 2011 census indicates that the sample is representative of Bhaluka subdistrict.

**Table 9:** Summary statistics for the sample.

	Mean	Std Dev
No. of family members	4.56	1.71
% households where highest education is		
Class 1–5	0.34	0.47
Class 6–10	0.46	0.5
Class 11–12	0.08	0.27
Beyond Class 12	0.05	0.22
No. of concrete liners in the pit	2.36	1.14
Annual household income (USD)	1245.61	1077.83
No. Women/household making toilet-management decisions	1.17	0.66
% households with non-farming income source	0.67	0.47
% households with brick walls	0.68	0.47
% households with metal sheet roof	0.97	0.17

Source: Authors' data

#### Current sludge emptying and transportation practices

Most pit latrines were installed around 4 years ago, with around 4–5 users per latrine. Around 20% of the sample had emptied their pit at least once, while the rest reported that they would have to have their pit emptied soon. Eighty-eight percent of all households reported that when their pits would be emptied next, a pit emptier would be hired. Ninety percent of households reported that sludge from their pits—when emptied—would be dumped in their backyards.

#### Estimating WTP for emptying and transportation service

Table 10 examines whether there is any evidence for an anchoring bias in the responses to bids, by tabulating the no/no, no/yes, yes/no and yes/yes responses for each of the four base bids. As the base bid amount increases, the number of no/no responses rises (column 1), and the number of yes/yes responses falls (column 4), as expected. When the base bid was BDT 400, 127 of the 286 households gave a response of yes/no, suggesting that for 44% of these household, the WTP lies between BDT 400–600. With a base bid of BDT 600, 144 of the 280 households gave a response of no/yes, indicating that the WTP of 51% of these households lies between BDT 400–600. For a base bid of BDT 700, 167 of the 282 households gave a response of no/no, suggesting the WTP of 59% of these households is less than BDT 600. Finally, when the base bid was 800, 180 of the 243 households gave a no/no response; the WTP of 74% of these households is less than BDT 700. These numbers suggest that the probability of accepting a base bid is not particularly sensitive to the starting point.

**Table 10:** Responses to the bid vectors.

			(1)	(2)	(3)	(4)
Base bid	HHs assigned		No/No	No/Yes	Yes/No	Yes/Yes
400		Intervals	0–300	300–400	400–600	>600
	286	No. of hh	27	91	127	41
		%	9.44	31.82	44.41	14.34
600		Intervals	0–400	400–600	600–700	>700
	280	No. of hh	73	144	38	25
		%	26.07	51.43	13.57	8.93
700		Intervals	0–600	600–700	700–800	>800
	282	No. of hh	167	82	21	12
		%	59.22	29.08	7.45	4.26
800		Intervals	0–700	700–800	800–1000	>1000
	243	No. of hh	180	31	27	5
		%	74.07	12.76	11.11	2.06

We estimate the WTP using: the value of the base bid; response to the base bid; the value of the follow-up bid; response to the follow-up bid; and the data on household characteristics. This uses information from 1,041 of the 1,091 households. Fifty observations were dropped by STATA as each of these had information perfectly collinear to another observation that was part of the analysis. We also bootstrap the estimates of WTP; 100 samples of 1,041 observations each were drawn with replacement. The estimate, and their confidence intervals, is presented in Table 11. The WTP for emptying and transportation service is estimated at ~ BDT 507.

**Table 11:** Household willingness to pay for emptying and transportation.

					Number of samples = 100 Number of obs/ sample = 100	
	Observed Coeff	Boostrap Std Err	z	P > |z|	Normal-based 95% CI	Bias-corrected CI
WTP	506.66	13.15	38.54	0.000	480.89–532.42	482.80–534.01

We also estimated a dichotomous choice model with no follow-up, using only the base bids and the responses, to check for an anchoring bias. This likelihood function is $\text{Pr(}y_{i}=1\;|x_{i})\;=\;\Phi \;\left(x_{i}^{\prime }\frac{\beta }{\sigma }-\;\frac{b_{i}}{\sigma }\right)$. After the estimates of $\hat{\beta }/\hat{\sigma }$ and $-1/\hat{\sigma }$ are obtained, $\hat{WTP}=\;-x_{i}^{\prime }\frac{\hat{\beta }/\hat{\sigma }}{-1/\hat{\sigma }}$. This gives an estimate of WTP to be BDT 435.69, and a standard error of 24.00. This is not statistically different from estimate of BDT 507 obtained using the follow-up questions.

## Discussion

This paper presents a methodology for identifying the actual per household costs of transportation of sludge for treatment from onsite latrines, using a cost-effective option, and links it to the households’ willingness to contribute to that cost. This allows the gap in financing of sludge management to be identified, to pave the way for considering alternative economic instruments to finance the gap.

For the case under consideration, we estimated that the cheapest cost per emptying event that can be achieved is BDT 1,088. The total costs of emptying and transport of fecal sludge in Bhaluka are significantly influenced by the choice of technology to be used. A combination of smaller units which can reach rural communities, and larger units for transporting sludge over longer distances seems to offer the best cost efficiencies. The operational component of the cost (including fuel and labor) comprise a significant portion of the costs of service provision (as much as seventy-five percent of the total costs of service provision in the case considered). Selecting appropriate technology combinations can lower total costs and reduce this percentage (to around sixty-three percent in the case considered).

We outlined a method by which we estimated that households in Bhaluka subdistrict are typically willing to pay up to BDT 507 for the emptying and transport away of the contents of their pit latrines. If willingness to pay remains unchanged then household contributions would currently cover only 47% of the cost of emptying and transport, leaving a deficit of BDT 581 per emptying event, equivalent to BDT 12 million per year (USD 154,500 per year) for Bhaluka subdistrict.

This deficit would most likely have to be met by local government and appears high. But another way to consider this is to think of emptying and transport as an ongoing *service* rather than as a one-off intervention, which is enjoyed on an ongoing basis by all households with pits. On Table 7 we calculate the *annual* cost per household with a pit, of the full emptying and transport service, to be BDT 292 (USD 4), assuming that every household would pay this amount irrespective of whether their pit was emptied in that year. In effect the service would then be provided on a subscription basis, similar to the payment of flat-rate water tariffs, or mobile phone contracts. The cost *per month per household* would be only BDT 24 (USD 0.31).

Converting payment regimes to regular payment of a predictable smaller amount could plausibly increase willingness and ability to pay and result in a higher proportion of emptying and transport costs being covered by households. In this case, a monthly fee of between USD 0.31 and 0.63 would be sufficient to cover the costs. This would enable public funds to be concentrated on the treatment element of the sanitation value chain. To date we are not aware of any similar analysis carried out in other settings with which these findings could be compared.

An alternative approach would be to invest funds in a marketing campaign to encourage households to construct and manage twin pit latrines thus negating the need for a centralized management service. Our household survey suggests that there is little interest in this; most households expressed a strong willingness to pay for a service which would result in the wastes being removed from the vicinity of the house. Additionally, observations suggest that few, if any, households have upgraded from a single to a twin-pit latrine in recent years. The high water tables characteristic of large parts of Bangladesh also mean that aerobic treatment potential inside pit latrines may be rather limited. Previous fieldwork suggested that pit contents retained high levels of pathogens, particularly helminth eggs, for a long period [7].

Expenditures on sanitation are often compared to household expenditures on mobile phones. In Bangladesh the penetration of mobile phone usage is high (an estimated 85% of the rural population report using a mobile phone) but most are pay-to-use, and many use community access platforms of various kinds. The mobile phone industry estimates that the annual revenue per subscriber is only USD 3.55 [46] suggesting that the required monthly fee for sanitation services would be only slightly higher than average monthly expenditure on communications.

This analysis covers the emptying and treatment portion of the sanitation value chain. For fully effective safe management of excreta, additional costs and benefits would need to be included in the analysis. On the cost side these include the capital, capital maintenance and operational costs of a treatment facility and on the benefits side the sale value of any treated product, in the form of agricultural inputs or energy. In general, there is more data available on the costs of treatment, but, as with emptying and transport, many of the available data probably underestimate ongoing operational costs. Furthermore, the value of treated products is difficult to estimate as they are strongly influenced by conditions in the downstream market. Currently for example, our research suggests that the sale value of agricultural products derived from human excreta may be depressed by the widespread presence of subsidized chemical fertilizers in the market in Bangladesh.

It seems likely therefore that some form of public subsidy would be required to support the full sanitation value chain, and may even be required to support the emptying and transport elements of the service. We expect this to be the case in other rural administrative areas where onsite sanitation is prevalent, and it needs to be managed. A subsidy is an economically justifiable option (though theoretically not the only option), since sanitation possesses a public good component in the form of health and environmental externalities.

While this study focused on the financial aspects of emptying and transport of fecal sludge there are numerous operational issues which would also need to be considered in implementing any management system. For example, we estimated that between 300 and 600 rural jobs could be created. Current practices of pit emptying are far from satisfactory, so a formalized system would need to include arrangements to secure the safety and well-being of this new cohort of pit emptying and transport workers, whether through regulation, contracts or other forms of incentives. Specific details of the location and nature of the treatment plants would also need to be assessed through a feasibility study.

## Conclusions

Countries such as Bangladesh which have been successful in terms of increasing access to sanitation in rural areas, now need to address the emerging challenge of fecal sludge management. This challenge is often seen as one of infrastructure provision, but perhaps a bigger challenge is working out how to establish a robust financial system which can cover the significant operating costs of a system for emptying and transport of fecal sludge to treatment. To date very little work has been done to establish the real costs of operating a rural sludge management system. This study set out to rectify that gap by designing a spreadsheet model to estimate actual costs and comparing this to willingness-to-pay as elicited through a purpose-designed household survey in Bhaluka district. The results are encouraging; while operating costs seem large when considered at district level, they appear to be affordable and appropriate when considered as part of a regular household budget. This study is the first step towards establishing a rural sanitation management service in Bangladesh on a sound financial footing.

More work is needed to establish the global factors which influence emptying and transport costs. Our model could be used to test costs in other geographies and markets and to establish the vulnerability of an emptying and transport service to variations in global prices for fuel, for instance. Nonetheless this analysis represents a significant step forward in our understanding of the real costs of operating rural sanitation services.

## References

[1] Fewtrell L, Kaufmann R B, Kay D, Enanoria W, Haller L, Colford J M Water, sanitation, and hygiene interventions to reduce diarrhoea in less developed countries: a systematic review and meta-analysis. *The Lancet Infectious Diseases* 2015 5 (1) 42–52 10.1016/S1473-3099(04)01253-8 15620560

[2] Prüss-Ustün A, Bartram J, Clasen T, Colford J M, Cumming O, Curtis V, et al Burden of disease from inadequate water, sanitation and hygiene in low- and middle-income settings: a retrospective analysis of data from 145 countries. *Tropical Medicine & International Health* 2014 19, pp. 894–905 24779548 10.1111/tmi.12329 PMC4255749

[3] Schmidt W P The elusive effect of water and sanitation on the global burden of disease. *Tropical Medicine and International Health*, 2014 19(5), pp. 522–527 10.1111/tmi.12286 24576060

[4] WHO Global costs and benefits of drinking-water supply and sanitation interventions to reach the MDG target and universal coverage 2012 World Health Organization Geneva http://apps.who.int/iris/bitstream/10665/75140/1/WHO_HSE_WSH_12.01_eng.pdf?ua=1 Accessed on 12 February 2016

[5] WHO & UNICEF Methodological note: Proposed indicator framework for monitoring SDG targets on drinking-water, sanitation, hygiene and wastewater prepared for the Inter Agency and Expert Group on Sustainable Development Goal indicators (IAEG-SDGs) 2015 http://www.wssinfo.org/fileadmin/user_upload/resources/Methodological-note-on-monitoring-SDG-targets-for-WASH-and-wastewater_WHO-UNICEF_8October2015_Final.pdf Accessed on 12 February 2016

[6] Heal G M Bundling biodiversity. *Journal of the European Economics Association*, 2003 1(2–3), pp. 553–60

[7] Balasubramanya S, Evans B, Ahmed R, Habib A, Asad N S M, Rahman M, et al Take it away: the need for designing fecal sludge disposal services for single-pit latrines. *Journal of Water*, *Sanitation and Hygiene for Development*, 2017 forthcoming

[8] Pattanayak S, Pfaff A Behavior, Environment, and Health in Developing Countries: Evaluation and Valuation. *Annual Review of Resource Economics*, 2009 1, pp. 183–217.

[9] Schaub-Jones, D Sanitation partnerships: beyond storage: on-site sanitation as an urban system. Building Partnerships for Development (BPD) Sanitation Series, 2008-11-8, 302.4.

[10] Evans B E, van der Voorden C and Peal A J Public Funding for Sanitation: The Many Faces of Sanitation Subsidies 2009 Water Supply and Sanitation Collaborative Council, Geneva, Switzerland. http://www.personal.leeds.ac.uk/~cenbee/Publications/Public_Funding_for_Sanitation_the_many_faces_of_sanitation_subsidies.pdf Accessed on 12 February 2016

[11] Bouabid A, Louis G E Capacity factor analysis for evaluating water and sanitation infrastructure choices for developing communities, Journal of Environmental Management, 2015 16, pp. 335–343 10.1016/j.jenvman.2015.07.012 26203872

[12] Fine P, Halperin R, Hadas E Economic considerations for wastewater upgrading alternatives: An Israeli test case. *Journal of Environmental Management*, 2006 78 (2), pp. 163–169, 10.1016/j.jenvman.2005.04.014 16115725

[13] Engin G O, Demir I Cost analysis of alternative methods for wastewater handling in small communities, *Journal of Environmental Management*, 2006 79(4), pp. 357–363, 10.1016/j.jenvman.2005.07.011 16307841

[14] Huang D B, Bader H P, Scheidegger R, Schertenleib R, Gujer W Confronting limitations: New solutions required for urban water management in Kunming City. *Journal of Environmental Management*, 2007 84(1), 49–61 10.1016/j.jenvman.2006.05.004 16857309

[15] Song Q, Wang Z, Li J Residents' behaviors, attitudes, and willingness to pay for recycling e-waste in Macau. *Journal of Environmental Management*, 2012 106, pp. 8–16 10.1016/j.jenvman.2012.03.036 22562006

[16] Blaine T W, Lichtkoppler F R, Jones K R, Zondag R H An assessment of household willingness to pay for curbside recycling: A comparison of payment card and referendum approaches. Journal of Environmental Management, 2005 76(1), pp. 15–22 10.1016/j.jenvman.2005.01.004 15854733

[17] Nixon H, Saphores J D M Financing electronic waste recycling Californian households’ willingness to pay advanced recycling fees. *Journal of Environmental Management*, 2007 84(4), pp. 547–559 10.1016/j.jenvman.2006.07.003 16979285

[18] Pek C P, Jamal O A choice experiment analysis for solid waste disposal option: A case study in Malaysia. *Journal of Environmental Management*, 2011 92(11), pp. 2993–3001, 10.1016/j.jenvman.2011.07.013 21820795

[19] Arimah B C Willingness to Pay for Improved Environmental Sanitation in a Nigerian City, Journal of Environmental Management, 1996 48(2), pp. 127–138.

[20] Afroz R, Hanaki K, Hasegawa-Kurisu K Willingness to pay for waste management improvement in Dhaka city, Bangladesh. *Journal of Environmental Management*, 2009 90(1), pp.492–503. 10.1016/j.jenvman.2007.12.012 18242819

[21] Kennedy-Walker R, Holderness T, Alderson D, Evans B E, Barr S Network modelling for road-based Fecal Sludge Management. *Proceedings of the Institution of Civil Engineers*: *Municipal Engineer*, 2014 167(3), 157–165.

[22] Whittington D, Briscoe J, Mu X, Barron W Estimating the Willingness to Pay for Water Services in Developing Countries: A Case Study of the Use of Contingent Valuation Surveys in Southern Haiti. *Economic Development and Cultural Change*, 1990 38(2), pp. 293–311.

[23] Briscoe J, Whittington D, Altaf M A, Decastro P F, Griffin C, Okorafor A, Smith V K The demand for water in rural-areas determinants and policy implications. *World Bank Research Observer*, 1993 8(1), pp.47–70. 12317957 10.1093/wbro/8.1.47

[24] Griffin C C, Briscoe J, Singh B, Ramasubban R, Bhatia R Contingent valuation and actual behavior—Predicting connections to new water-systems in the State of Kerala, India. *World Bank Economic Review*, 1995 9(3), 373–395.

[25] Whittington D, Lauria D T, Wright A M, Choe K, Hughes J F, Swarna V Household demand for improved sanitation services in Kumasi, Ghana: A contingent valuation study. *Water Resources Research*, 1993 29(6), pp. 1539–1560.

[26] Yusuf S A, Salimonuand K K, Ojo O T Determinants of Willingness to Pay for Improved Household Solid Waste Management in Oyo State, Nigeria. Medwell Journals, *Research Journal of Applied Sciences*, 2007 2 (3), pp.233 239.

[27] Balasubramanya S, Evans B, Ahmed R, Habib A, Asad N S M, Vuong L et al Pump it up: making single-pit emptying safe in rural Bangladesh 2016 6(3):456–464 *Journal of Water*, *Sanitation and Hygiene for Development*.

[28] Ministry of Planning, Government of Bangladesh Community Report: Mymensingh Zila-June 2012. Population and Housing Census 2011, Bangladesh Bureau of Statistics, Statistics and Informatics Division. Accessed on Feb 13, 2016 at http://203.112.218.66/WebTestApplication/userfiles/Image/Census2011/Dhaka/Mymensingh/Mymensingh%20at%20a%20glance.pdf

[29] World Bank Water and Sanitation Program Economic Impacts of Inadequate Sanitation in Bangladesh 2012 World Bank Water and Sanitation Program. http://www.wsp.org/sites/wsp.org/files/publications/WSP-ESI-Bangladesh-Report.pdf Accessed on 12 February 2016

[30] Morgan P Toilets That Make Compost: Low-cost, sanitary toilets that produce valuable compost for crops in an African context.2007 Aquamor: Harare, Zimbabwe Stockholm Environment Institute, EcoSanRes Programme

[31] Shivendra B T, and Ramajaru H K Impact of Onsite Sanitation System on Groundwater in Different Geological Settings of Peri Urban Areas International Conference on Water Resources Coastal and Ocean Engineering Aquatic Procedia 2015 4 pp 1162–1172

[32] Dzwairo B, Hoko Z, Love D, Guzha E Assessment of the impacts of pit latrines on groundwater quality in rural areas: A case study from Marondera district, Zimbabwe *Physics and Chemistry of the Earth* 2006 31 pp 779–788

[33] Evans B E, Balasubramanya S and Hardy R Fecal Sludge emptying and transportation modelling tool; Calculation of transportation and emptying costs for pit latrines 2016 University of Leeds, International Water Management Institute https://github.com/Barbaraevansuk/Fecal-sludge-empty-and-transport-costs/blob/master/Costing.Spreadsheet.-.Bhaluka.Upazila.Mymensignh.Zila.Dhaka.Divison.Bangladesh.-.FINAL.xlsx Accessed 23rd February 2016

[34] Khan I Using Discount Rate for Water Sector Planning in Bangladesh under Climatic Changes. *International Journal of Business Quantitative Economics and Applied Management Research*, 2015 2(2), pp 110–117

[35] Boyle K J Contingent Valuation in Practice in Champ P, Boyle K J and Brown T C Eds A Primer on non-market Valuation 2003 Kiuwer Academic Publishers ISBN: 9789400708266

[36] Carson R T, Groves T Incentives and Informational Properties of Preference Questions. *Environmental and Resource Economics*, 2007 37(1), pp. 181–210.

[37] Hanemann W M, Loomis J, Kanninen B Statistical Efficiency of Double-Bound Dichotomous Choice Contingent Valuation. *American Journal of Agricultural Economics*, 1991 73(1), pp. 1255–1263.

[38] Green D, Jacowitz K, Kahnemann D, McFadden D Referendum Contingent Valuation, Anchoring, and Willingness to Pay for Public Goods. *Resource and Energy Economics*, 1998 20(2), pp. 85–116.

[39] Herriges J A, Shogren J F Starting Point Bias in Dichotomous Choice Valuation with Follow-up Questioning. *Journal of Environmental Economics and Management*, 1996 30(1), pp. 112–131.

[40] Alberini A Optimal Designs for Discrete Choice Contingent Valuation Surveys: Single-Bound, Double-Bound, and Bivariate Models. *Journal of Environmental Economics and Management*, 1995 28(3), pp. 287–306.

[41] Alberini A Willingness-to-pay for Discrete Choice Contingent Valuation Survey Data. *Land Economics*, 1995 71(1), pp. 83–9.

[42] Boyle K J, MacDonald H F, Cheng H, McCollum D W Bid Design and Ye Saying in Single Bounded, Dichotomous-Choice Questions. *Land Economics*, 1998 74(1), pp. 49–64

[43] Cooper J C Optimal Bid Selection for Dichotomous Choice Contingent Valuation Surveys. *Journal of Environmental Economics and Management*, 1993 24(1), pp. 25–40

[44] López-Feldman. Introduction to contingent valuation using Stata. 2013 MPRA paper 41018.https://mpra.ub.uni-muenchen.de/41018/2/MPRA_paper_41018.pdf

[45] Delevande A, Xavier G, McKenzie D J Measuring subjective expectations in developing countries: a critical review and new evidence. *Journal of Development Economics*, 2011 94(2), pp. 151–163

[46] GSMA Intelligence Country Overview: Bangladesh 2015 https://gsmaintelligence.com/research/?file=140820-bangladesh.pdf&download Accesssed on 12 February 2016

